# Imaging of Spinal Metastatic Disease

**DOI:** 10.1155/2011/769753

**Published:** 2011-11-03

**Authors:** Lubdha M. Shah, Karen L. Salzman

**Affiliations:** Neuroradiology Division, Department of Radiology, School of Medicine, The University of Utah, 1A71 SOM, 50 N. Medical Drive, Salt Lake City, UT 84132, USA

## Abstract

Metastases to the spine can involve the bone, epidural space, leptomeninges, and spinal cord. The spine is the third most common site for metastatic disease, following the lung and the liver. Approximately 60–70% of patients with systemic cancer will have spinal metastasis. *Materials/Methods*. This is a review of the imaging techniques and typical imaging appearances of spinal metastatic disease. *Conclusions*. Awareness of the different manifestations of spinal metastatic disease is essential as the spine is the most common site of osseous metastatic disease. Imaging modalities have complimentary roles in the evaluation of spinal metastatic disease. CT best delineates osseous integrity, while MRI is better at assessing soft tissue involvement. Physiologic properties, particularly in treated disease, can be evaluated with other imaging modalities such as FDG PET and advanced MRI sequences. Imaging plays a fundamental role in not only diagnosis but also treatment planning of spinal metastatic disease.

## 1. Introduction

Metastases to the spine can involve the bone, epidural space, leptomeninges, and spinal cord. The spine is the third most common site for metastatic disease, following the lung and the liver [1] and the most common osseous site [2]. Approximately 60–70% of patients with systemic cancer will have spinal metastasis. Fortunately, only 10% of these patients are symptomatic. The frequency with which spine metastases are detected varies considerably with the type of primary tumor. Common tumors with a high rate of metastasis to bone include tumors of the breast (72%), prostate (84%), thyroid (50%), lung (31%), kidney (37%), and pancreas (33%). Together, these account for more than 80% of primary tumors in patients presenting with metastases [3, 4]. The extradural lesions account for up to 95% of spinal lesions and can be divided into pure epidural lesions and those originating from the vertebra extending to the epidural space and subsequently impinging on the thecal sac [5]. The thoracic spine is most commonly involved. Intradural extramedullary and intramedullary seeding of systemic cancer is unusual, accounting for 5–6% and 0.5–1% of spinal metastases, respectively. In general, the prognosis for patients presenting with bone metastases is poor [6].

## 2. Imaging Techniques and Pitfalls

### 2.1. Radiography

**Figure 1:**
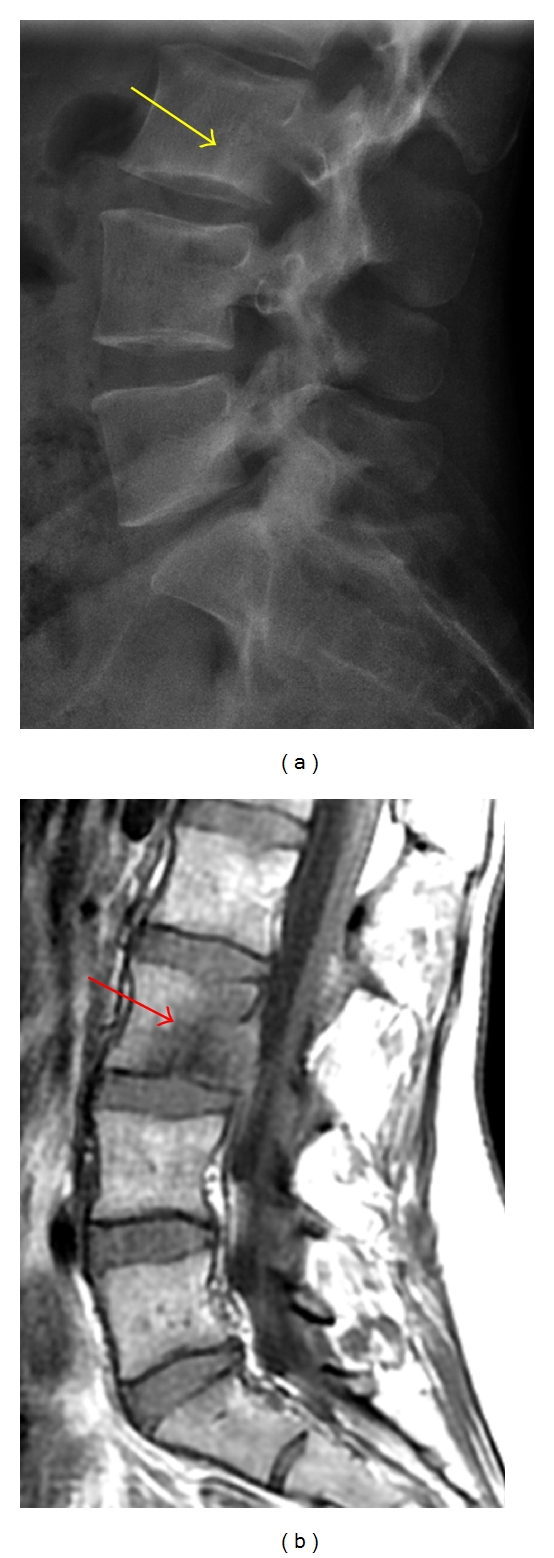
(a) Lateral radiograph is poor at delineating the L3 vertebral body metastatic lesion, which appears as a faint lucency with a subtle sclerotic margin (yellow arrow). (b) This lesion is better seen on the sagittal T1-weighted MRI as an ill-defined hypointensity (red arrow) within the L3 marrow.

Radiographs are an ubiquitous modality for the evaluation of back or neck pain in the setting of trauma or in the evaluation of degenerative changes. However, X-rays necessitate a 1 cm diameter mass and 50% bone mineral loss at minimum for detection. Up to 40% of lesions will be unidentified by X-rays, presenting false-negative results [7] (Figure 1). Radiography may be a crude assessment of the risk of pathologic fracture, which is said to be high if 50% of the cortex is destroyed by tumor [6]. Epidural lesions may demonstrate osseous erosion along the posterior vertebral body margin or pedicles. Rarely, metastases may cause scalloping of the adjacent bone.

### 2.2. Nuclear Medicine

**Figure 2:**
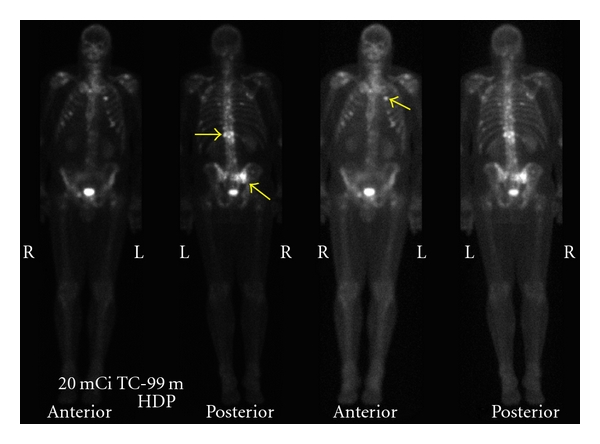
Anterior and posterior bone scan planar images reveal multiple foci of increased radionuclide uptake, not only in the thoracic and lumbar spine but also in the ribs and sacrum (yellow arrows).

Nuclear medicine bone scans (bone scintigraphy) have been the standard initial imaging method for screening for skeletal metastases. Tracer accumulates in the reactive new bone that is formed in response to the lesion (Figure 2). The amount of accumulation is sensitive to the level of blood flow. Although most metastatic lesions are “hot,” lesions that are cold due to the complete absence of reactive bone or poor blood flow may be encountered in particularly aggressive metastases. Diffuse accumulation of tracer throughout the skeleton (super scan) may occasionally occur in disseminated skeletal disease, leading to the false impression of a normal scan. This is most common with prostate carcinoma. False-negative studies are most common with multiple myeloma (up to 25% of cases), leukemia, and anaplastic carcinomas. Single-photon-emission computed tomography (SPECT) scanning. SPECT imaging has improved both the sensitivity and the specificity of bone scanning [8], particularly with larger lesions and cortical involvement. Because tracer accumulation may occur at any skeletal site with an elevated rate of bone turnover, radionuclide uptake may be nonspecific and may accompany trauma, infection, arthropathy, or osteopenia of disuse. In a patient with a known primary tumor, a scan showing multiple lesions strongly suggests metastases. However, only 50% of solitary foci represent metastases, even in patients with cancer [6]. Positive scans should be correlated with contemporaneous radiographs because of this lack of specificity.

 [^18^F]fluoro-2-deoxy-d-glucose positron emission tomography (FDG-PET) can detect increased glucose metabolism of neoplastic cells nested in the bone marrow, making it a sensitive method for assessment of bone and bone marrow metastases. [^18^F]-FDG PET alone and [^18^F]-FDG PET registered with CT have a reported sensitivity of 74% and 98%, respectively, in the detection of spinal metastasis [9]. [^18^F]-FDG PET is reportedly more sensitive than bone scintigraphy in patients with lung cancer and lymphoma and was shown to detect early bone marrow involvement before cortical changes could be seen by bone scintigraphy [10, 11]. [^18^F]-FDG PET is more sensitive for detection of osteolytic metastasis than of osteoblastic metastasis [12]. Schmitz et al. demonstrated that [^18^F]-FDG PET is able to differentiate between osteoporotic and malignant vertebral compression fractures in patients with [^18^F]-FDG-avid tumors [13].

### 2.3. Computed Tomography

**Figure 3:**
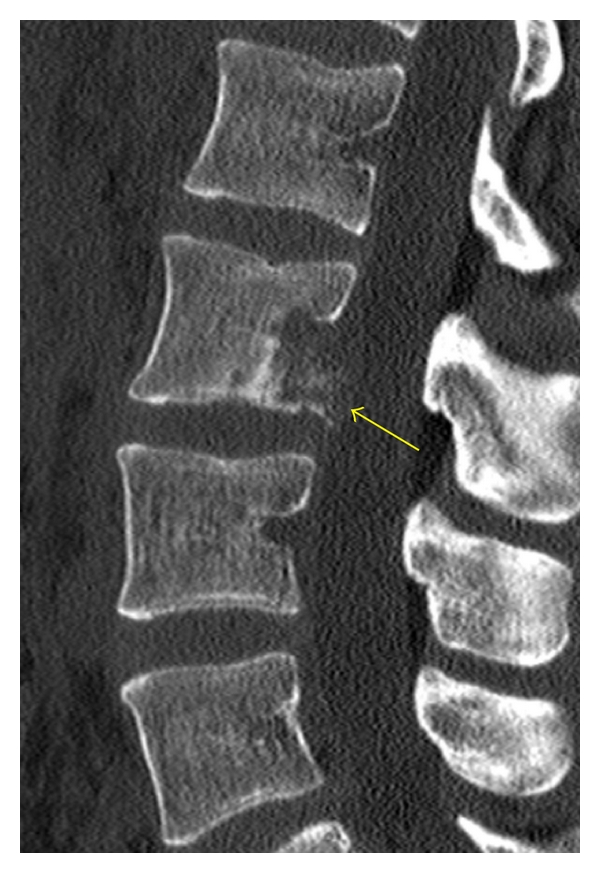
Sagittal CT reformation in bone algorithm depicts a cortical break in the posterior cortex of the L3 vertebral body (yellow arrow) due to a metastatic focus.

**Figure 4:**
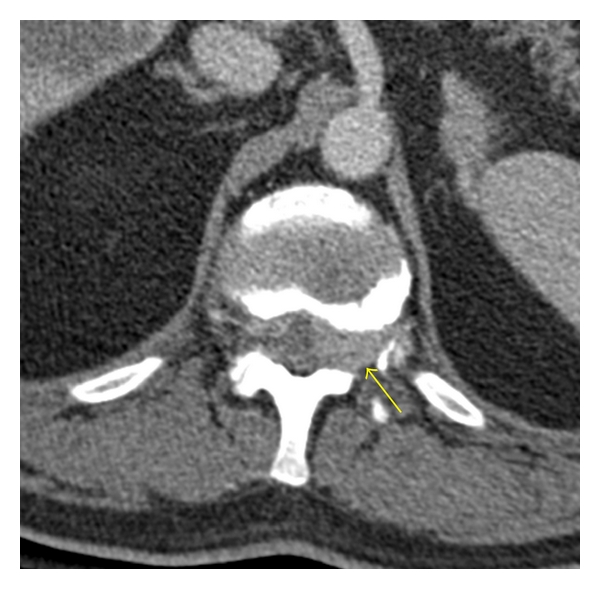
Axial CT in soft tissue algorithm displays slightly hyperdense soft tissue in the ventrolateral epidural space filling the left neural foramen (yellow arrow) and causing mass effect on the thecal sac.

Computed tomography (CT) scans can recognize a bony metastatic lesion up to 6 months earlier than an X-ray [7]. CT gives superb osseous delineation and enables detection of cortical destruction (Figure 3). An epidural mass may present as amorphous soft tissue displacing the thecal sac or filling the neural foramen (Figure 4).

Although 16/64-row-MDCT provides excellent image quality and a high spatial resolution in the assessment of bony structures, metastatic lesions without significant bone destruction may be missed. Buhmann et al. found the diagnostic accuracy of MRI (98.7%) to be significantly superior to 16/64-row-MDCT (88.8%) for the detection of osseous metastases [14]. Sensitivity was significantly lower for MDCT (66.2%) than for MRI (98.5%) (*P* < 0.0001). The specificity was not significantly different for both methods (MDCT: 99.3%; MRI: 98.9%). One disadvantage of CT is the beaming hardening artifact that obscures the adjacent soft tissues and bones. Another disadvantage of CT is that cortical destruction may be difficult to detect when osteoporosis or degenerative changes occur [15]. Finally, there is an inherent associated risk of radiation exposure from the CT.

### 2.4. CT Myelography

CT myelography is a helpful technique in those patients who cannot undergo an MRI (e.g., patients with pacemakers, extreme claustrophobia). It allows assessment of osseous integrity as well as the thecal sac contents and has the added benefit of allowing CSF sampling at the same time as the diagnostic test is performed. Soft tissue characterization is better performed with MRI. CT myelography may show metastatic disease as thickened nerve roots, subarachnoid masses, and/or blockage of the subarachnoid space.

### 2.5. Magnetic Resonance Imaging

**Figure 5:**
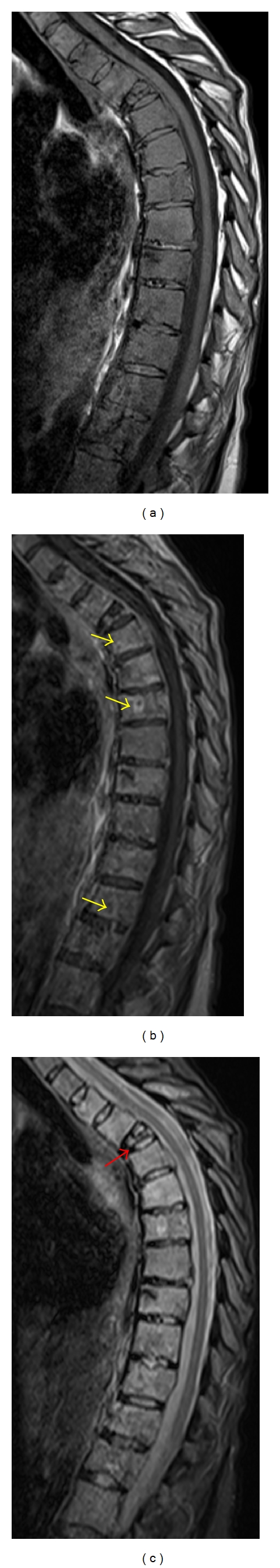
Sagittal T1-weighted MR image (a) of the thoracic spine illustrates diffuse marrow hypointensity, which is slightly hypointense relative to the discs. Given the diffuse marrow involvement, it may be difficult to discern this marrow abnormality. Gadolinium-enhanced T1-weighted MR image (b) depicts multiple heterogeneously enhancing lesions (yellow arrows). The STIR MR image (c) shows abnormally increased signal in the posterior elements and the vertebral bodies. A compression fracture is seen in the upper thoracic spine (red arrow).

Unlike CT, which detects bony abnormalities, particularly cortical destruction, magnetic resonance (MR) imaging can detect early bone marrow deposits (Figure 5). Studies have shown that MR imaging has a significant impact on spinal tumor evaluation [16]. Specific relevant diagnostic information that can be gleaned from MR imaging of the spine includes the diagnosis of metastasis, the characterization of the levels of involvement, and the diagnosis of any associated cord compression. Both bony involvement and neural compression from epidural tumor are demonstrable by MR imaging. MRI is the only imaging technique that allows direct visualization of bone marrow and its components with high spatial resolution. The combination of unenhanced T1-weighted-spin echo- and STIR-sequences have shown to be most useful for the detection of bone marrow abnormalities and are able to discriminate benign from malignant bone marrow changes. Because of its sensitivity to bone marrow abnormalities, MRI may serve to guide biopsy of areas of abnormal signal intensity [17].

### 2.6. MR Sequences

Normal marrow contains both fat and water (yellow marrow 80% fat, but also 15% water, and red marrow 40% fat and 40% water). In infiltrative disorders, fat disappears in a diffuse, disseminated or solitary way. Sequences displaying differences between fat and water signal are thus useful.

### 2.7. T1-Weighted Spin-Echo (SE) Sequences

Fat has a shorter signal than water and the highest signal. Thus, fatty marrow containing 80% fat exhibits a high signal and any focal lesion showing a lower signal is easy to detect. This explains why this sequence is very useful and usually the first used. Hematopoietic marrow, containing water but also fat, is hypointense to fat, but hyperintense to normal muscles. At 1.5 T, a marrow signal which is hypointense to the muscles and discs in the spine is abnormal with an accuracy of 94% and 98%, respectively [18]. The study by Zhao et al. showed a higher diagnostic accuracy using signal intensity of muscle (89%) versus disk (78%) at 3 T field strength [19]. Replacement of the bone marrow always appears hypointense relative to normal marrow on T1-weighted images [17, 20]; however, this hypointensity is nonspecific. Extensive replacement of the vertebral bone marrow may initially create the impression of a normal study (Figure 5).

### 2.8. T2-Weighted Sequences

**Figure 6:**
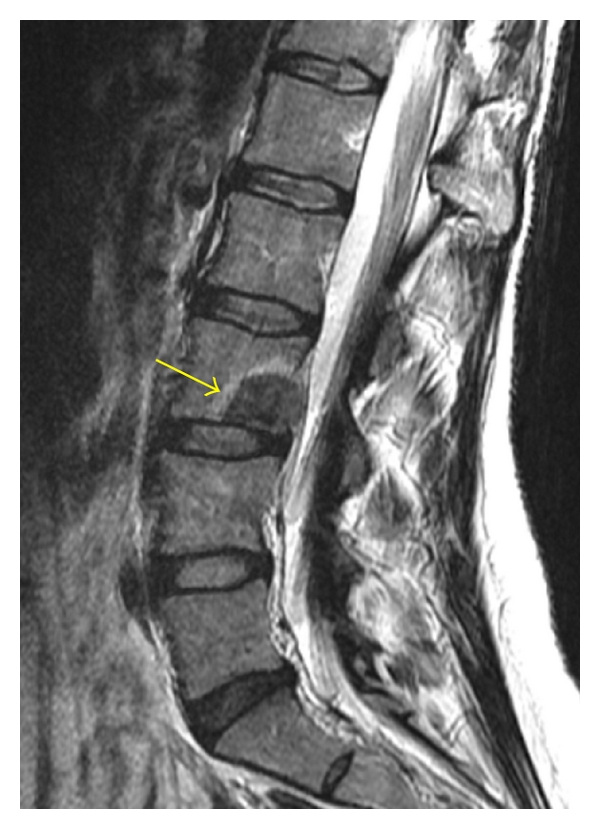
Sagittal T2-weighted MR image depicts the “halo sign” with a hypointense metastatic lesion and a surrounding hyperintense rim in the L3 vertebral body (yellow arrow).

Conventional spin echo (SE) and fast spin echo T2 sequences have been shown to detect the same number of lesions [21] with the latter being a much more rapid sequence. On T2-weighted images, metastatic lesions are usually much brighter than bone marrow, due to their high water content. Metastases often (but not consistently) have a rim of bright T2 signal around them (a halo sign) [22] (Figure 6). The halo sign and diffuse signal hyperintensity were shown to be a strong indicator of metastatic disease (sensitivity, 75%; specificity, 99.5%). The bull's-eye sign (focus of high signal intensity in the center of an osseous lesion) is a specific indicator of normal hematopoietic marrow (sensitivity, 95%; specificity, 99.5%) [22].

**Figure 7:**
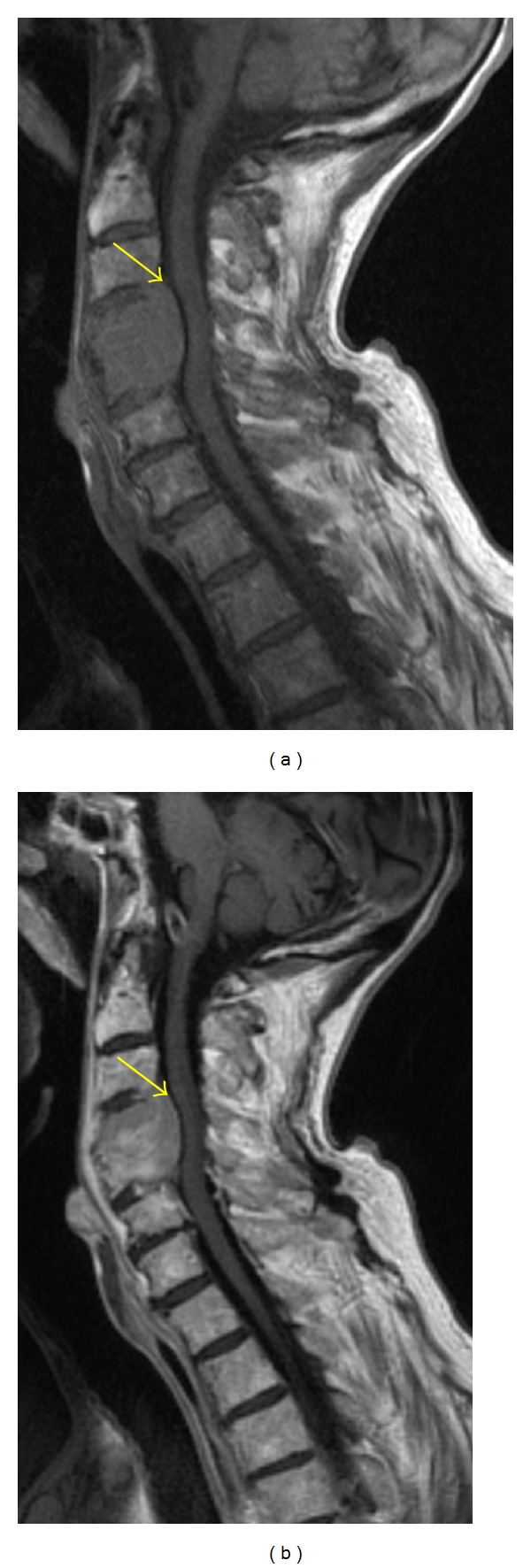
Sagittal T1-weighted MR image (a) shows a hypointense expansile lesion involving the C4 and C5 vertebral bodies with extension into the ventral epidural space (yellow arrow). The lesion enhances homogeneously on postcontrast T1-weighted MR (b); however, the degree of normal marrow enhancement is similar to that of the metastatic myeloma lesion.

Contrast is typically administered in standard tumor imaging as it allows for identification of intramedullary and intradural extramedullary abnormalities and extradural lesions (particularly in the epidural space) that may result in compression of the spinal cord and alter treatment [23] (Figure 7). However, on T1-weighted sequences, enhancing metastases may become isointense with normal bone marrow and become obscured. Sequences that suppress the signal intensity of normal fatty bone marrow allow clear identification of the enhancing metastatic foci [24]. T1 postcontrast with fat saturation can increase the conspicuity of enhancing marrow lesions by suppressing the background bright fatty marrow signal.

### 2.9. Fat Suppression Techniques

A 180 inversion pulse is used initially for short tau inversion recovery (STIR) sequences [25]. The inversion time is chosen to cancel the signal of fat. This sequence can be obtained on any MR unit, but it is unfortunately time consuming and only a limited number of slices can be acquired. This can be overcome by using fast STIR sequences. 

Although the conspicuousness of lesions is similar on fat-saturation T2-weighted and STIR images, the former sequence has several practical advantages, including acquisition of more slices per unit time and improved tissue specificity [25]. The combination of T1-weighted and either fat-saturation T2-weighted or STIR images is highly effective for the evaluation of bone marrow lesions. On fat-suppressed, T1-weighted images, metastases demonstrate mixed-to-high signal intensity, whereas nonneoplastic lesions have low signal intensity [26]. Fat saturation techniques are particularly sensitive to susceptibility artifact from spinal hardware.

### 2.10. Diffusion-Weighted Imaging (DWI)

DWI evaluates the tissue-specific molecular diffusion of protons. In tissues with high cell densities (neoplasm), a decreased ADC can be expected due to restricted diffusion according to an exaggerated amount of intra- and intercellular membranes (i.e., diffusion barriers). The utility of DWI on differentiating benign from metastatic spinal lesions is controversial in the literature. One study using DWI found all benign vertebral compression fractures from hypo- to isointense to adjacent normal vertebral bodies and pathologic compression fractures were hyperintense to normal vertebral bodies [20]. However, Castillo et al. show in their series of 15 patients that DWI of the spine showed no advantage in the detection and characterization of vertebral metastases as compared with noncontrast T1-weighted imaging, but was considered superior to T2-weighted imaging [27]. Others have demonstrated that rather than qualitative assessment, the quantitative evaluation of the ADC in vertebral bodies may be an objective and comparable parameter for differentiating malignant from benign vertebral tissue [28]. 

Unfortunately, MRI often cannot distinguish among changes that are due to treatment, fracture, and tumor. Hanna et al. compared MRI scans with histologic specimens at 21 sites, 7 of which contained tumor and 14 of which did not. For all of the tumor positive sites, abnormalities were revealed on MRI scans. However, for the sites shown to be free of tumor, there was a significant false-positive rate, presumably because tumor could not be distinguished from the effects of treatment [29]. DWI sequences may show decreased signal intensity of metastatic disease of the vertebral marrow with successful treatment [30]. 

### 2.11. Whole Body MRI

Whole-body MRI represents a new alternative to the stepwise multimodality concept for the detection of metastatic disease, multiple myeloma, and lymphoma of the bone with high diagnostic accuracy [24]. The introduction of a rolling platform mounted on top of a conventional MRI examination table facilitates whole body MR imaging and—with the use of fast gradient echo, T1-weighted, and STIR-imaging techniques—allows whole body imaging within less than one hour. With the development of parallel imaging techniques in combination with global matrix coil concepts, acquisition time is reduced substantially without compromises in spatial resolution, enabling the implementation of more complex and flexible examination protocols.

## 3. Pathology

Bone destruction, secondary to metastases, is caused by the activation of osteoclasts rather than by the direct destruction of bone by tumor cells. Mundy and Yoneda proposed that cells from the primary site migrate or through the process of neovascularization attach to the basement membrane of the vessel wall and produce proteolytic enzymes that disrupt the basement membrane [31]. The tumor cells then migrate to a distant site hematogenously attaching to the basement membrane of the vessel wall using proteolytic enzymes (integrins/cadherins). After disrupting the receptor site basement membrane, they migrate into the substance of the distal host tissue. Producing the chemotactic factors, as well as RANK ligand, these cells stimulate osteoclast activity to produce bone resorption. A feedback relationship, such as that present in myeloma cells, produces continued osteoclast stimulation for bone resorption and tumor cell growth. This continued growth and survival of the metastatic cells progressively destroys cancellous and cortical bone at the distant osseous site.

**Figure 8:**
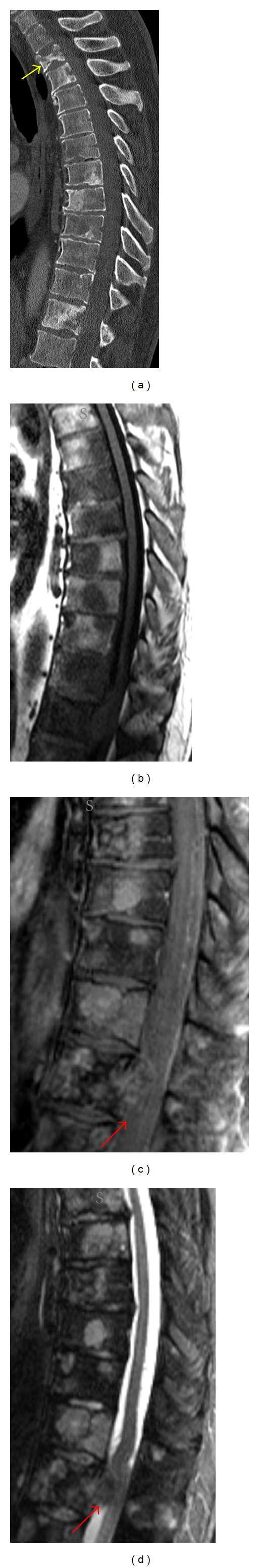
Sagittal CT reformation (a) shows multiple lytic and blastic metastatic breast cancer lesions in the thoracic spine with a compression fracture in the upper thoracic spine (yellow arrow). Sagittal T1-weighted image (b) shows multiple hypointense lesions, many of which enhance on the postcontrast T1-weighted MR (c). The STIR image (d) depicts both hyperintense (lytic) and hypointense (blastic) lesions. A mildly enhancing epidural component compresses the thecal sac (red arrows).

Primary tumors which typically have lytic spinal metastases are breast, lung, kidney, thyroid, oropharyngeal, melanoma, adrenal, and uterus. Breast and lung cancer may also show mixed lytic and sclerotic lesions, which are seen with ovarian, testicular, and cervical carcinomas (Figure 8). Lytic lesions involve the posterior cortex almost always with destruction of the posterior cortex and pedicle. If the discs appear brighter than bone on T1-weighted MR, it is concerning for diffuse marrow infiltration. Lytic lesions typically exhibit diffuse enhancement. Progressive sclerosis of a lytic focus generally indicates a positive response. However, if there is persistently low T1 signal in marrow after therapy, this may indicate either active tumor or fibrosis. Functional techniques such as DWI and in phase/opposed phase are being investigated as potential MR sequences for such diagnostic dilemmas [32]. 

**Figure 9:**
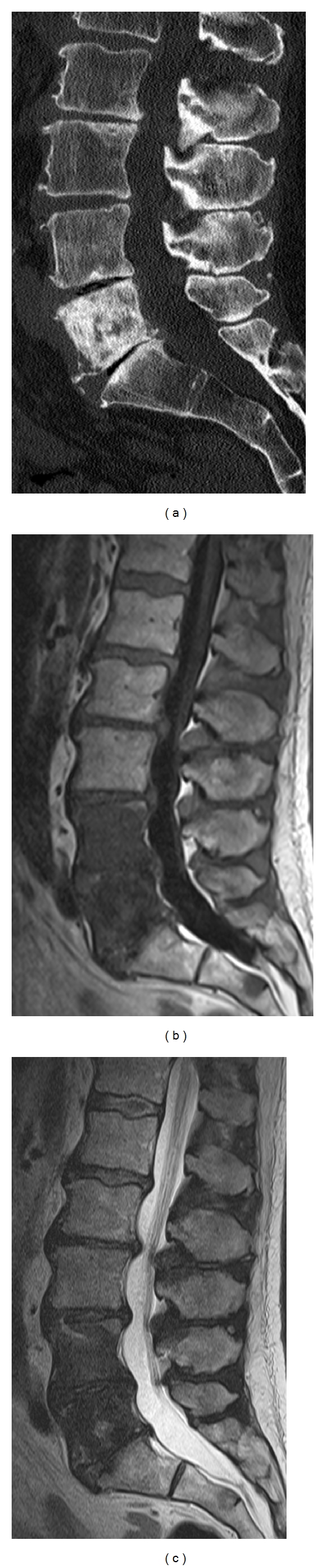
Sagittal CT reformation of the lumbar spines (a) shows a large sclerotic lesion nearly completely involving the L5 vertebral body. Sagittal T1-weighted (b) and T2-weighted (c) images show abnormal hypointense marrow signal in not only the L5 vertebral body but also the L4 vertebral body, corresponding to blastic metastatic lesions.

Prostate, bladder, nasopharynx, medulloblastoma, neuroblastoma, and bronchial carcinoid primaries commonly have blastic-appearing spinal metastases. The areas of sclerosis may be nodular or mottled in appearance. Occasionally, there may be diffuse areas of increased density on radiographs and CT with corresponding hypointensity on all MR sequences (Figure 9). Blastic metastases tend to destroy the posterior cortex and involve the pedicle. It is important to assess for an associated paraspinal or epidural component. Tumor may spread into the anterior epidural space with sparing of the meningovertebral ligament, resulting in the “draped curtain sign.” The enhancement pattern is variable depending on the degree of sclerosis. Fat-suppression increases the conspicuity of enhancing lesions. It may be difficult to evaluate the therapeutic response of sclerotic lesions as tumor progression with osteolytic conversion appears similar to fading, which is seen in good response. 

Hematogeneous spread of metastatic disease is far more frequent than lymphatic spread or direct invasion. The venous route, especially Batson's paravertebral plexus, appears to be more important than the arterial route. The distribution of Batson's venous plexus, as well as the overall skeletal vascularity, results in a predilection for hematogenous spread to the axial skeleton and the proximal long bones.

Metastases may reach the skeleton by direct invasion from the primary tumor or by extension from a secondary site, such as a lymph node. True lymphatic spread to the skeleton is rare. Direct invasion is usually accompanied by a detectable soft tissue mass, an unusual feature of metastases that occur by hematogenous spread. 

However, hematogeneous spread of metastatic disease is far more frequent than lymphatic spread or direct invasion. The venous route, especially Batson's paravertebral plexus, appears to be more important than the arterial route. The distribution of Batson's venous plexus, as well as the overall skeletal vascularity, results in a predilection for hematogenous spread to the axial skeleton and the proximal long bones.

### 3.1. Disease Progression

Symptomatic spinal cord compression is seen in approximately 10%–20% of cases with metastatic spinal involvement [2]. Research has shown that noncontrast T1-weighted images are probably the most useful type of images in adult patients with clinically suspected cord compression, because vertebral metastases are most often appreciated with this MR imaging sequence [33–35]. A study comparing different MR protocols found unenhanced T1-weighted images may be sufficient for evaluation of possible cord compression and guiding radiation treatment [36]. 

**Figure 10:**
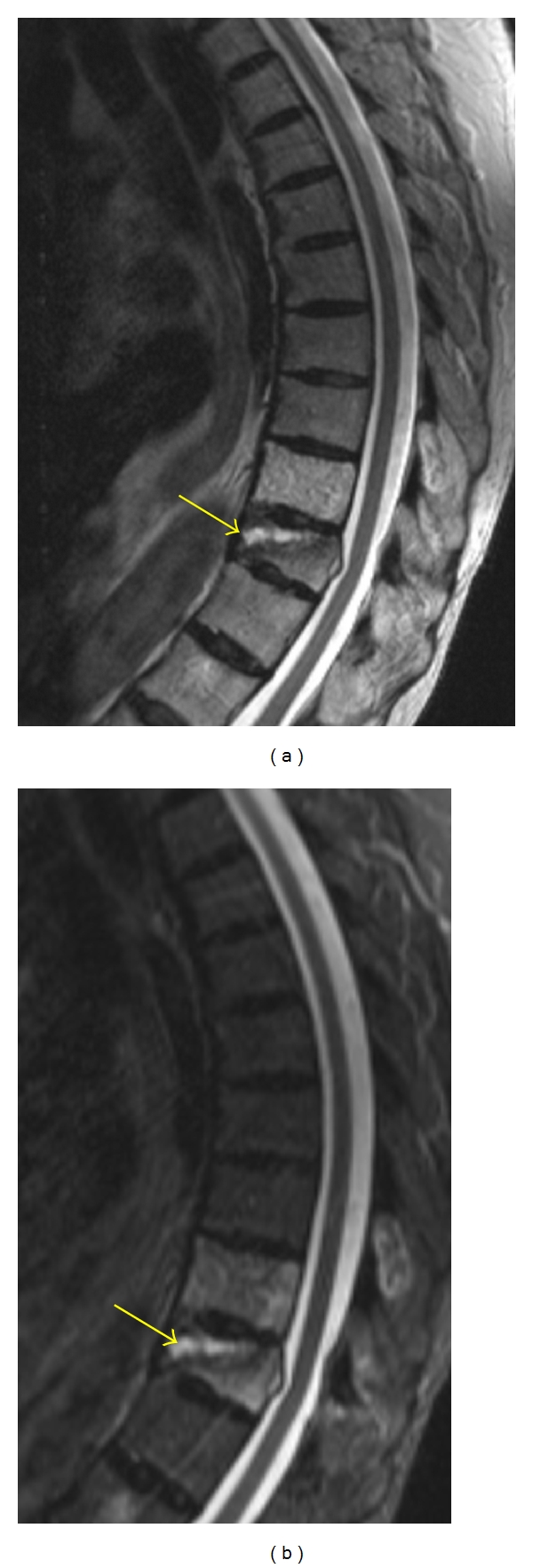
Sagittal T2-weighted (a) and STIR (b) MR images reveal abnormal hyperintense signal in a lower thoracic vertebral body due to fracture-related edema. A “fluid sign” is demonstrated in this vertebral body (yellow arrow), which is characteristic of a benign osteoporotic fracture.

Benign compression fractures and malignant lesions can show a considerable overlap. Edema in a benign compression fracture in the acute phase replaces the normal marrow, resulting in hypointensity on T1-weighted images and hyperintensity on T2-weighted images. The vertebral body with benign fracture may show enhancement. The morphology of bone marrow replacement may be helpful for prediction of the benign or pathologic cause of a fracture. Conventional MRI features have been cited to suggest pathologic fracture: a convex posterior border of the vertebral body, abnormal signal intensity of the pedicle or posterior element, an encasing epidural mass, a focal paraspinal mass, and other spinal metastases [37]. Paravertebral soft-tissue masses and infiltration of posterior elements are the most reliable signs of a malignant fracture. MR imaging findings suggestive of acute osteoporotic compression fractures include a low-signal-intensity band on T1- and T2-weighted images, spared normal bone marrow signal intensity of the vertebral body, retropulsion of a posterior bone fragment, and multiple compression fractures [37]. The MR fluid sign has been described in avascular necrosis of the vertebral body [38, 39] and is a common finding in acute and subacute benign osteoporotic vertebral fractures [39]. Up to 40% of these fractures may show the fluid sign [40] (Figure 10). Morphologic criteria may accurately predict benign from malignant fractures of the spine in up to 94% of cases [41].

Quantitative ADC mapping, instead of qualitative diffusion-weighted imaging, may provide valuable information in differentiating benign vertebral fractures from metastatic lesions [42]. Lower ADC values have been demonstrated in pathologic fractures [42].

Vertebral metastases may invade the epidural space by direct extension from adjacent bone through the posterior longitudinal ligament, by extension through the intervertebral foramina, by hematogenous dissemination, or very rarely by lymphatic infiltration. Involvement of the epidural space may result in compression of the spinal cord or cauda equina or in radiculopathy because of compression of nerve roots [43]. The neurologic symptoms due to the soft tissue material impinging upon the epidural venous plexus results in venous hypertension and vasogenic edema [44]. The epidural tumor [44] and/or the vertebral collapse [45] may have direct mass effect in the spinal cord leading to neurologic deterioration. However, occasionally the involvement may be asymptomatic. 

**Figure 11:**
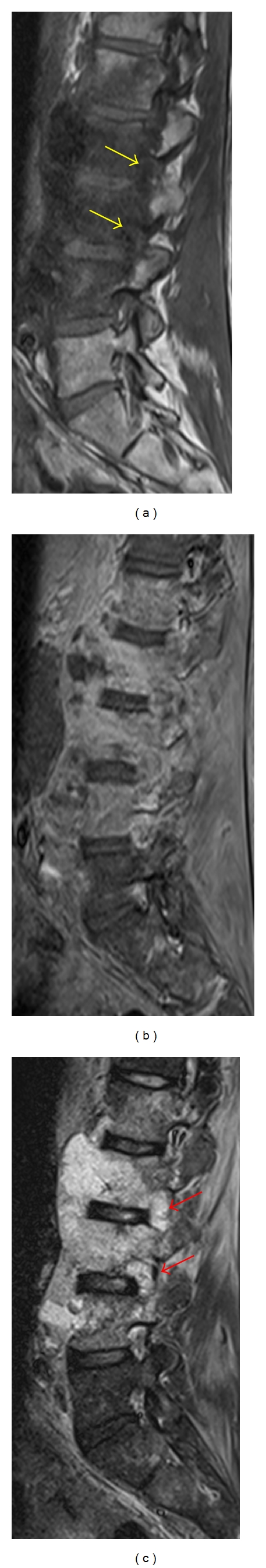
Sagittal T1-weighted MR image (a) shows abnormal hypointensity related to invasion by a paraspinal mass (spindle cell sarcoma). The cortical margins of the pedicles are attenuated (yellow arrows) in keeping with erosive changes of the mass invading through the neural foramina into the lateral epidural space. The postcontrast T1-weighted MR image with fat-saturation (b) shows heterogeneous enhancement of the mass. Marrow infiltration and neuroforaminal involvement (red arrows) is well seen as hyperintense signal on the STIR MR image (c).

Bone destruction is seen often, up to 86% of the time [46], at the level of epidural tumor involvement (Figure 11). Pedicular erosion on radiographs predicts epidural disease in 31% of cases [47]. Epidural metastasis is often contiguous with a vertebral body lesion. The meningovertebral ligament is characteristically spared giving the “draped curtain” appearance. Lesions tend to be T1 hypointense, T2 hyperintense, and avidly enhancing. In cases on spinal epidural lymphoma, the spinal column may actually be spared. Contrast enhancement is helpful in delineating the extent of tumor and may help in outlining regions of spinal cord compression [35]. This is particularly useful in the cervical and thoracic spine where there is relative paucity of epidural fat and prominent ligaments, which typically increase the conspicuity of epidural lesions. The thoracic spine is more often (*∼*60%) involved in neoplastic epidural spinal cord compression as compared to *∼*30% in the lumbosacral spine [48]. This predilection may be due to the reduced potential space available for tumor to expand. 

Leptomeningeal carcinomatosis (LC) has an incidence as high as 4–15% in patients with solitary tumors, 5–15% in patients with leukemia and lymphoma, and 1-2% in patients with primary brain tumors [40, 49]. In autopsy studies, the rate has been estimated to be 19% in patients with cancer and neurologic signs [50]. It is most commonly found in breast carcinoma, lung carcinoma, and melanoma in adults and hematogenous malignancies and primitive neuroectodermal tumor (PNET) in children. Less commonly, prostate cancer can spread to the leptomeninges. Melanoma and lung cancer have the highest rates of spread to the leptomeninges at 20% and 11%, respectively [51, 52]. The routes of dissemination to the meninges include hematogenously though Batson's venous plexus or arterial spread, direct extension from contiguous foci of tumor, and perineural or perivascular migration from systemic tumors [53, 54]. Tumor cells in the CSF are carried throughout the neural axis, particularly the base of the brain and the dorsal spinal cord surface and cauda equina [54].

Spinal symptoms of LC include extremity weakness (greater lower extremity involvement), dermatomal or segmental sensory loss, and neck and/or back pain [49, 55]. While there are clinical signs and radiologic findings that strongly suggest LC, most cases are diagnosed by CSF cytology or leptomeningeal biopsy. As the diagnostic accuracy of lumbar puncture (LP) is only 50–60% after a single LP and 90% after 3 LPs, MRI is considered complementary and can be invaluable, detecting up to 50% of cases with false-negative LPs. It is important to note that CSF levels of protein, glucose, and malignant cells vary at different levels of the neuraxis even without an obstructive lesion [56]. 

**Figure 12:**
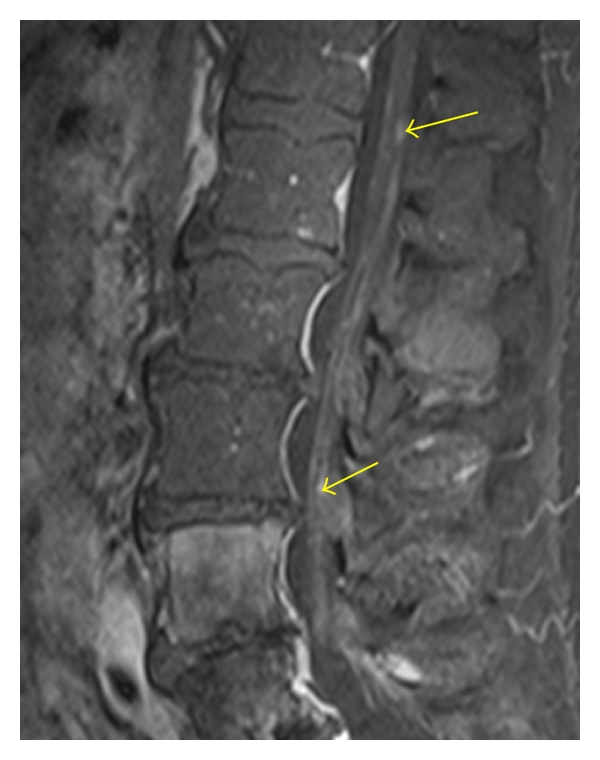
Sagittal contrast-enhanced T1-weighted MR image with fat saturation reveals multiple small enhancing lesions (yellow arrows) along the cauda equina, related to leptomeningeal carcinomatosis in a patient with breast cancer.

**Figure 13:**
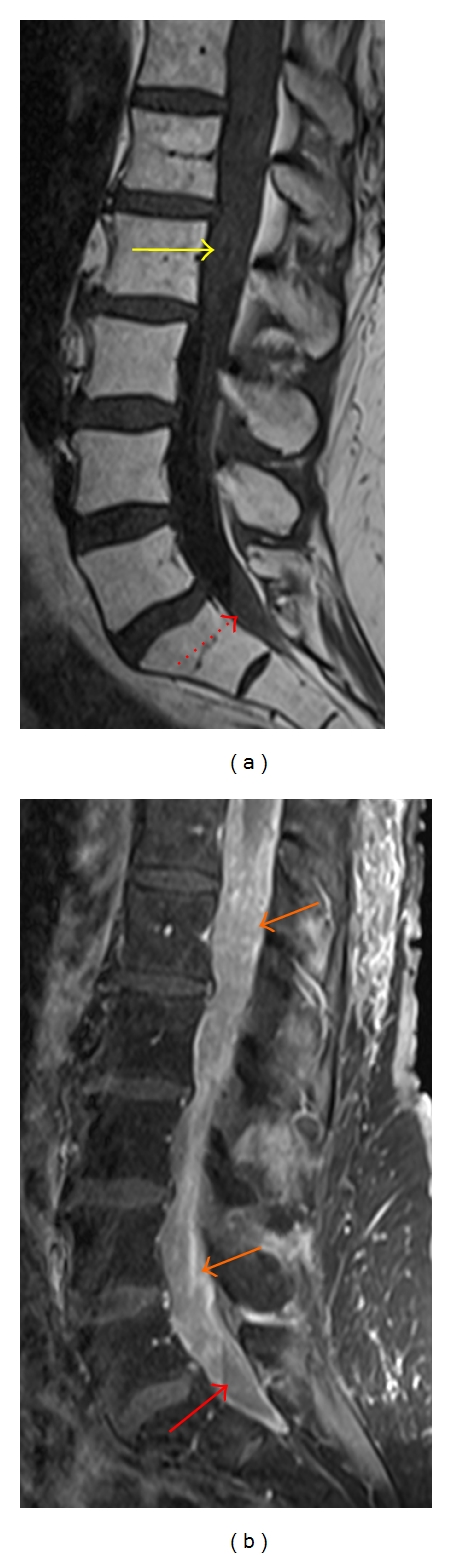
Sagittal T1-weighted MR image (a) shows a hazy appearance of the subarachnoid space (yellow arrow) in the distal thecal sac. Fluid layers dependently in the thecal sac from blood products and/or proteinaceous debris (red arrows). Sagittal enhanced T1-weighted MR image (b) with fat-saturation shows diffuse enhancement of the CSF as well as thick sheet-like enhancement of the cauda equina (orange arrows) related to metastatic melanoma.

Imaging the whole neuraxis is required as LC can involve the entire CNS. Detection of CNS enhancement indicates a poor prognosis [57]. MRI and CT may demonstrate multiple masses within the subarachnoid space, hydrocephalus without a discernible cause, or diffuse leptomeningeal enhancement. The latter enhancement pattern has been referred to as sugar icing or “zuckerguss” and can be found in the brain, spine, or both. The nerve roots may be thickened, particularly of the cauda equina, and there may be subarachnoid nodules (Figure 12). CSF enhancement is uncommon but when seen indicates massive tumor that coats the surface of the CNS (Figure 13). Radioisotope CSF flow studies may be performed to prior to intrathecal chemotherapy in order to ensure no obstruction of CSF flow and homogeneous distribution of the chemotherapeutic agent. 

Gadolinium-enhanced T1-weighted MRI is superior to contrast-enhanced CT in detecting abnormal leptomeningeal enhancement and the complications of meningitis including cerebritis and ventriculitis. Sze et al. reported the efficacy with which gadolinum-enhanced MRI of the spine can detect even small lesions in the intradural extramedullary space [58]. However, other studies have shown that the evaluation of leptomeningeal metastasis with MRI and CT modalities may have a high incidence of false-negative studies, 89% (31 of 35) by CT and 24% (4 of 17) by MRI [59]. The literature reports a difference in sensitivity between solid tumors and hematologic malignancies, with one study reporting a sensitivity of 90% in patients with solid tumors but only 55% in patients with lymphoma and leukemia [60]. 

Intramedullary spinal metastatic lesions are exceedingly rare, representing only 8.5% of all CNS metastases. They affect an estimated 0.5–2% of patients with cancer and comprise 1–3% of all intramedullary spinal cord tumors [61–63]. Intramedullary spinal cord metastases are due to lung cancer in 50–60% of cases with small cell lung cancer comprising 50% of these cases [61–63]. The presence of an intramedullary spinal metastatic lesion suggests an advanced biologically aggressively form of cancer. The metastatic deposits are usually solitary but may be multifocal in 15% of cases [63]. 

The mechanism of metastatic spread from the primary tumor to the spinal cord is thought to be hematogenous via an arterial route in the majority of cases. In some cases, there may be retrograde spread from the vertebral venous plexus or directly from the CSF via perivascular spaces in patients with LC. Extension from an adjacent neoplasm directly though the dura or by perineural spread has also been speculated. 

**Figure 14:**
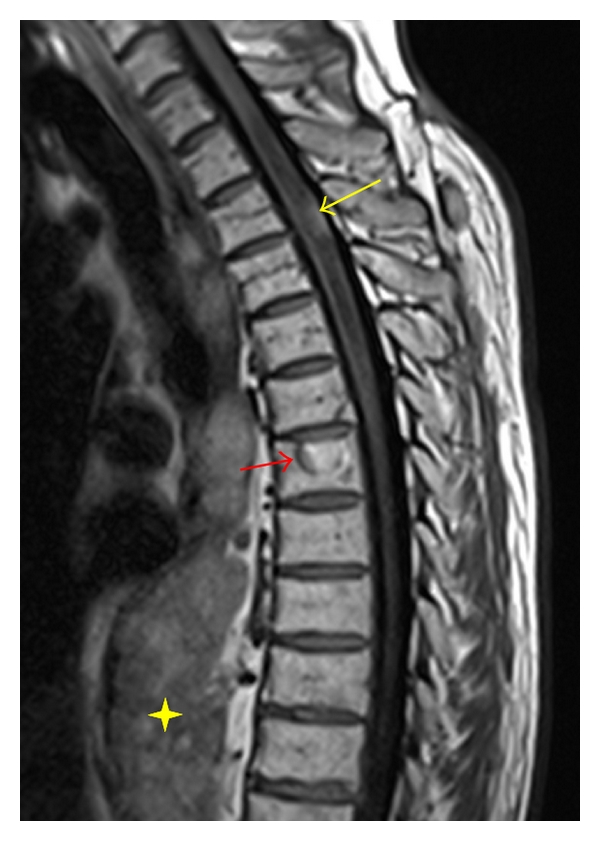
Sagittal T1-weighted MR image reveals an intramedullary hyperintense lesion in the dorsal upper thoracic cord from a melanoma metastasis (yellow arrow). There is extensive surrounding hypointensity due to spinal cord edema. Additionally, there is a T1 hyperintense vertebral body metastatic lesion (red arrow). Large lobulated paraspinal masses are also noted (yellow star).

The characteristic MRI finding is a small, intensely enhancing lesion, typically less than 1.5 cm, with extensive associated edema. There may be an enlargement of the cord. T1 hyperintensity may be detected with melanoma spinal cord metastases (Figure 14). Hemorrhagic intramedullary metastases may demonstrate hypointensity on T2 and T2* gradient-recalled echo images. 

### 3.2. Spinal Metastatic Disease Mimics

**Figure 15:**
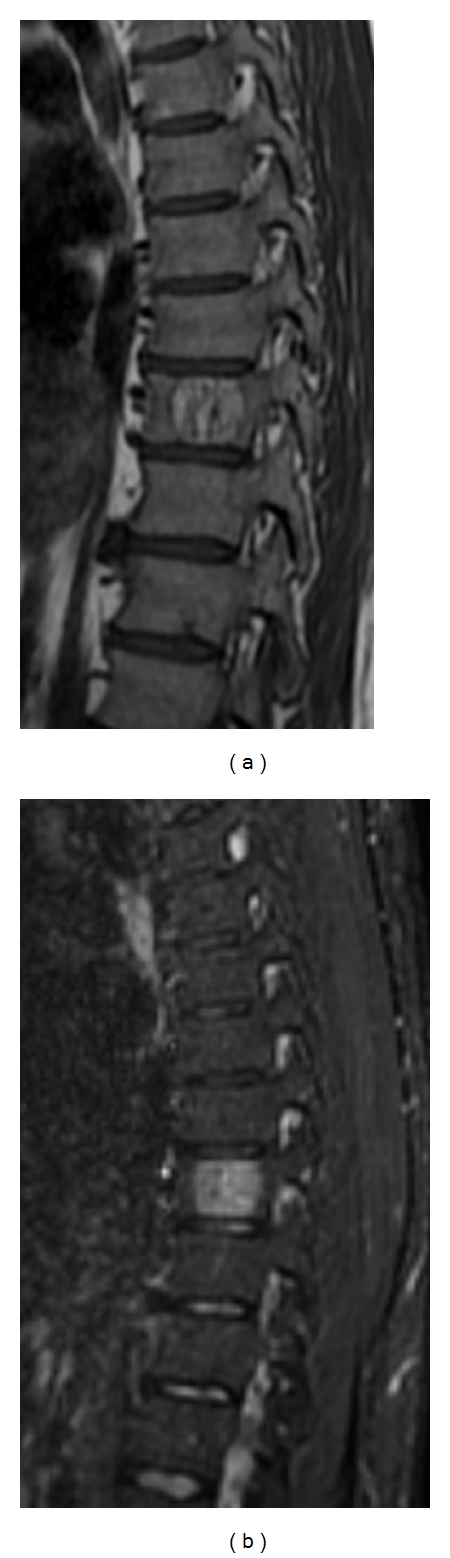
Sagittal T1-weighted (a) and STIR (b) MR images depict a well-circumscribed lesion in a mid thoracic vertebral body. The hyperintense signal and linear hypointensities, which correspond to thickened trabeculae, are characteristic of a benign hemangioma.

The imaging differential diagnosis of vertebral body metastasis would include benign hemangioma, discogenic endplate changes, and discitis-osteomyelitis. Vertebral hemangiomas are typically well-circumscribed, benign vascular tumors, which are T1 hyperintense (Figure 15). These lesions may be dark or bright on STIR sequences dependent on the proportion of fatty and vascular elements. The coarse vertical trabeculae resemble corduroy or honeycomb of radiographs. Internal trabeculae may be subtle on MRI and may be better delineated on CT in cases of “atypical” hemangiomas. Given the vascular component of these lesions, enhancement is common. Type 1 fibrovascular discogenic endplate changes display T1 hypointensity, T2 and STIR hyperintensity, and enhancement. The signal changes parallel the endplates, and the disc space usually shows loss of height and low T2 signal due to degeneration. Similarly, acute intravertebral disc herniation or Schmorl's node will demonstrate signal abnormality related to edema, including T1 hypointensity and T2/STIR hyperintensity. In discitis-osteomyelitis, there are endplate erosions with intradisc fluid and patchy enhancement. The adjacent endplates demonstrate abnormal fluid marrow signal and enhancement. Osseous metastases typically do not cross the disc space from one vertebral body to the next. The avascular disc is resistant to tumor invasion. 

Other lesions that may involve the epidural space include epidural hematoma and epidural phlegmon/abscess. Epidural hematoma is generally contained in the “less common” spectrum of intraspinal, extradural lesions, particularly in the absence of sentinel events such as surgical manipulation or trauma. Spontaneous epidural hematomas are rare but have been reported in patients on anticoagulation [64], those with vascular malformations [65] and in pregnancy [66]. A rare case report of spinal epidural hematoma associated with unsuspected metastatic lung cancer has been described [61]. Depending on the status of the hemoglobin and its intracellular versus extracellular location, the MR features will vary. In the acute stage, it will be hyperdense on CT with progressive hypodensity as the blood products evolve. The dorsal epidural space is more often involved with the multisegmental fluid collection. There may be peripheral enhancement; however, focal enhancement should be concerning for active extravasation [67].

Epidural abscess or phlegmon is often seen in association with spondylodiscitis. Hematogenous dissemination from gastrointenstinal, genitourinary, cutaneous, lung, and cardiac infection sources is also possible. Direct inoculation from iatrogenic procedures or trauma is an additional etiology of spinal epidural abscess. The posterior epidural space is involved more often than the anterior epidural space. Peripheral enhancement with a necrotic core (abscess) or diffuse enhancement (phlegmon) may be seen on contrast-enhanced T1-weighted MRI. Fat saturation techniques make lesions in the epidural space more evident by suppressing the normal epidural fat. 

When evaluating an MRI with an intramedullary enhancing lesion, the differential considerations include demyelinating disease, granulomatous process, cord infarction, cord vascular malformation, and primary cord tumor (such as astrocytoma, ependymoma, hemangioblastoma). Clinical history may provide clues to narrow the differential diagnosis. For instance, CSF positive for oligoclonal bands and waxing and waning symptoms in a young female adult would favor a demyelinating process. Osseous metastases in a patient with a known primary malignancy would make an enhancing intramedullary lesion more suspicious for an intramedullary metastasis. Prominent vascular flow voids along the cord surface in addition to intramedullary edema are helpful in determining if the lesion is a spinal arteriovenous malformation. 

## 4. Conclusion

Awareness of the different manifestations of spinal metastatic disease is essential as the spine is the most common site of osseous metastatic disease. Imaging modalities have complimentary roles in the evaluation of spinal metastatic disease. CT best delineates osseous integrity while MRI is better at assessing soft tissue involvement. Physiologic properties, particularly in treated disease, can be evaluated with other imaging modalities such as FDG PET and advanced MRI sequences. Imaging plays a fundamental role in not only diagnosis but also treatment planning of spinal metastatic disease.
